# The Education of the Hospital Officer

**Published:** 1915-03-13

**Authors:** George Watts

**Affiliations:** Secretary, City of London Hospital for Diseases of the Chest.


### THE EDUCATION OF THE HOSPITAL OFFICER.

### Examinations and a Three Years' Course.

By GEORGE WATTS, Secretary, City of London Hospital for Diseases of the Chest.

Education, though strictly the educing or drawing out of the latent powers of an individual, has acquired in general use a more comprehensive meaning. It implies the correction of qualities unfavourable to attainment of success in any selected field of work as well as the acquirement of knowledge. In its application to the hospital officer it assumes this wider significance, embracing instruction of an intellectual character and also the cultivation of certain personal qualities. Within those limits there remains ample scope for individuality, but the man who possesses all the requisite faculties, even though in only average degree, will be successful where the man lacking in one, although possessing the others in high degree, will fail.

To those who appreciate the special nature of hospital work, the standard suggested will not appear too exacting. The goal is the combination of all the qualifications, but if we fail to attain to the ideal, we may yet achieve that degree of efficiency which escapes spoiling by some shortcoming. The complexity of hospital life—the inter-relation of various departments, the diversified character of the material requirements, the dependence upon public approbation—is what constitutes its charm, but it adds also to the responsibilities. It is evident that in the chief officer, or officers, there is need of powers of organisation and co-ordination, of a not inconsiderable measure of business ability, and, not least, of qualities such as tact (a varying personal attribute), sensibility to the standpoint of others (not always evident in the strong-minded organiser), and a general ability to make matters go smoothly without sacrifice of control or efficiency. Similar qualities are requisite, though in varying degree, in junior officers. In these considerations will be found the need of the standard suggested. The education of the hospital officer is not, then, amongst the simplest of problems.

The aim of this article, although it has so far applied to officers of all classes, is to draw attention particularly to the needs in the secretarial department. It will be admitted that under the voluntary system that department possesses an importance which is hardly capable of over-statement. To the chief officer, be he styled secretary, superintendent, house governor, director, or manager, falls a responsibility in respect of administration, as the representative of the governing body, and of the inauguration of means of obtaining funds, which is of the utmost importance. It may be that his responsibility is not so much expressed as that he enjoys, in the performance of his duties, a scope which is almost without limit, and opportunities which are full of interest and possibility.

Outside the established professions, few occupations provide so congenial a sphere of work. The chief officer may be accused of being a Jack-of-all-trades, but that will not detract from the value of his work if he have the energy and devotion to learn what are the principles of the various negotiations and business affairs which come under his observation, and he has the satisfaction of knowing that an exercise of ordinary shrewdness is not without value in relation to many of the technical matters which arise in connection with his work. To adopt Emerson's phrase, the chief officer's sphere is assuredly "not a station," but most certainly "a progress."

The advantage of a scheme of education does not consist entirely in its value as a means of training. From the point of view of the junior officer its importance lies in the creation of a standard of efficiency, by means of examination, which will assist him in obtaining promotion. In the hospital world the general impression is that the absence of any systematic training is the principal reason why committees making appointments to senior posts have, in some instances, apparently attached small value to previous experience. It may well be that uncertainty of promotion tends to have a prejudicial effect on the *personnel* of the secretarial department; it cannot be expected that the most suitable junior clerks can be obtained under such conditions.

The fundamental difficulty in the way of providing the necessary machinery for promotion—paradox though it be—arises from the very virtues of the voluntary system.

The conditions of employment of hospital officers are peculiar. They are a comparatively small body of specialised workers, having no security of position nor assurance of promotion; but as compensations to those drawbacks they know that efficiency, combined with good conduct, makes their posts sure; their positions are not precarious as is the case in occupations dependent upon commercial conditions. They would probably, for these reasons, be unwilling to change to any other pursuit, if they were reasonably assured of adequate facilities for promotion. The fact is that they enjoy an environment so free and natural that it has always a stimulating effect; that advantage is, however, more evident to senior officers. Although it is true that they must not expect to enjoy, side by side with the fine simplicity of the voluntary system, all that pertains to a rigid bureaucracy, the opportunities of promotion at present appear less than they might reasonably be, and, consequently, some pining for a bureaucratic and systemised control may be understood. Although there is an increasingly close relation between the hospitals, there is no facility for a free interchange as regards appointments.

The education of the hospital officer is therefore not of itself sufficient to ensure success; but it is probable that the organisation of a scheme of education, and the recognition of its utility by the governing bodies of the hospitals, would facilitate the movement of officers to different hospitals not only in senior posts, but in junior ones also.

The extent of the tuition of junior officers within the ordinary routine of hospital work varies considerably according to the size and character of the hospitals in which they are employed and the degree of interest which the chief officers take in the hospital service in general. It is rather in the moulding of personal qualities that hospital experience plays the more important part, and it is in supplement of this that the formation of a definite scheme of education is valuable.

The subjects on which the energetic junior officer will welcome tuition may be stated as follows:—

Office methods and committee work.
The inter-relation of officers and departments.
Medical qualifications.
Financial and statistical matters.
Constructional matters : upkeep of buildings.
History and theoretical points : relation of voluntary institutions to public assistance.

These headings could well be elaborated, and a scheme on these lines has, in fact, already been inaugurated by the Incorporated Association of Hospital Officers, in the curriculum embracing instruction in three grades. The writer does not feel at liberty to give any official account, but a pronouncement of the aims of the Association in this respect may well be expected when the scheme has developed sufficiently to be productive of the results desired. It is obvious that, when that time arrives, the full fruit of the scheme can be obtained only by the greatest publicity. It may be mentioned that the three grades of tuition take the form of lectures spread over a three years' course, with an examination in the highest grade annually. It is not suggested that examination for entrance to hospital service is desirable. It may well be argued that the defects of the competitive system—generally recognised—would be most marked in relation to hospital work, where personal qualities are of considerable importance.

As with all things at this time, the war has created a condition of affairs which has made it very difficult to apply the scheme, but the writer is optimistic enough to believe that the start made will result in an adequate system and provide a criterion of capacity which will justify the reasonable claims of junior officers to promotion to senior posts. The above points naturally lead one to consider how far the most important of the qualifications necessary to a successful chief officer may be possessed by a man not trained in the hospital world. On this head it appears fairly evident, however, that the most suitable personal qualities, unless associated with great adaptability, will not compensate for lack of special training.

The difficulty of the question of obtaining recognition of the junior officer's claims arises from the fact that election by committee has certain obvious disadvantages, notably the tendency for outside influences to exercise considerable weight, and the fact that it does not always fall to the most active members of the committee, who have the best opportunity of appreciation of the needs, to carry their views. It can be stated with certainty that the untrained man must be a less known quantity than the tried man who can produce evidence of efficiency in some branches of the work. It may be accepted without question that the well-being of hospital work would gain immeasurably by its development as a definite service, through the resulting attraction of the most suitable men to the junior posts.

It is probably beyond question that if preliminary experience of business methods could be required of juniors entering, the hospital service would gain immeasurably thereby; but this would postpone the age of entry to eighteen or twenty years, and the present rates of salary for junior posts plainly precludes that course.

The absence of regularity in promotion operates with particular hardship in the case of some officers. There is considerable difference between hospitals of varying size as regards the filling of appointments. The post of secretary at hospitals generally is, as regards remuneration, much superior to that of the junior posts, even including assistant secretaryships. In hospitals of moderate size, where this difference obtains in less degree, the secretarial post is often filled by promotion in the hospital; but in the smallest hospitals, again, where the junior officers are very young, men are appointed from other hospitals. The number of senior posts being few, a man attached to a small hospital, and having reached a reasonable maximum in a junior post, may find it extremely difficult to obtain a better post in a larger hospital.

If, in addition to the developments suggested, some greater uniformity of remuneration in the different institutions could be achieved, without the stifling effect of a fixed scale, the principal requirements of a stable and satisfactory service would, in the opinion of the writer, be practically met. Hospital officers as a class are enthusiastic in their views, and devoted to their work—a natural result of the free conditions of the voluntary system. It is probable that these same officers placed under a bureaucratic system would suffer loss of initiative through the absence of the influences which foster individuality.

An important matter closely related to the developments suggested is that of pensions for hospital employees. It is a valuable evidence that the voluntary system is not so hopelessly heterogeneous that the aspirations of years may now crystallise. It was announced in the last annual report of the Council of the Incorporated Association of Hospital Officers, in May last, that the Executive Committee of the King's Fund, who had stated, in response to a memorial from the Association in December 1910, that they would be in favour at some future time of holding an inquiry into this question, were prepared to make the inquiry, and were ascertaining whether it would meet with the approval of the hospital committees and their assistants by supplying information. The carrying through of these proposals would go a long way towards systematising the hospital service and encouraging the entrance to it of the most suitable men.

